# Population-based epidemiology of intensive care: critical importance of ascertainment of residency status

**DOI:** 10.1186/cc2947

**Published:** 2004-10-15

**Authors:** Kevin B Laupland

**Affiliations:** 1 Assistant Professor, Department of Critical Care Medicine, Department of Medicine, and Department of Pathology & Laboratory Medicine, University of Calgary, Calgary Health Region, and Calgary Laboratory Services, Calgary, Alberta, Canada

**Keywords:** incidence, intensive care unit, mortality, population-based

## Abstract

**Introduction:**

Few studies evaluating the epidemiology of critical illness have used strict population-based designs that exclude subjects external to the base population. The objective of this study was to evaluate the potential effects of inclusion of nonresidents in population-based studies in intensive care.

**Methods:**

A population-based cohort study including all adults admitted to Calgary Health Region (CHR) multidisciplinary and cardiovascular surgical intensive care units (ICUs) between 1 May 1999 and 30 April 2003 was conducted. A comparison of patients resident and nonresident in the base population was then performed.

**Results:**

A total of 12,193 adult patients had at least one admission to an ICU; 7767 (63.7%) were CHR residents, for an incidence of 263.7 per 100,000 per year. Male CHR residents were at significant increased risk for ICU admission as compared with females (330.5 per 100,000 versus 198.2 per 100,000; relative risk, 1.67; 95% confidence interval, 1.59–1.74; *P *< 0.0001), as were CHR residents aged 65 years and older as compared with younger patients (1719.9 per 100,000 versus 238.7 per 100,000; relative risk, 7.21; 95% confidence interval, 6.95–7.47; *P *< 0.0001). The mortality rate was significantly lower among non-CHR residents (12.7%) as compared with CHR residents (20.0%; *P *< 0.0001). Logistic regression modeling identified CHR residency as an independent risk factor for death (odds ratio, 1.4; 95% confidence interval, 1.2–1.5; *P *< 0.0001).

**Conclusion:**

This study provides information on the incidence of and demographic risk factors for admission to ICUs in a defined population. Inclusion of patients that are nonresident in base study populations may lead to gross errors in determination of the occurrence and outcomes of critical illness.

## Introduction

Knowledge of the occurrence of and determinants of critical illness is important for establishing its burden and the risk factors for acquisition to guide wise allocation of limited healthcare and research resources. Population-based cohort studies that strictly include all episodes of disease occurring in residents of a geographically defined region are commonly accepted as the optimal design for such purposes [1-3]. However, these designs have rarely been used in the critical care medical literature [4-6]. Studies attempting to evaluate the distribution and determinants of critical illness typically have been case series reported from academic tertiary care referral hospitals [7-9]. Multicentered studies that include intensive care units (ICUs) in different regions and/or countries have less commonly been performed [10-13].

A major limitation to these institution-based studies is that if the population at risk is unknown, then incidence rates may not be calculated. Furthermore, if these studies focus on tertiary care centers and fail to include critically ill patients admitted to ICUs in other hospitals, a biased assessment of disease occurrence and severity may occur [14]. This may still be problematic even if all ICUs in a defined geographic region are included if investigators do not exclude patients nonresident in that base population from analysis [15]. Although referral bias has been shown to be of major importance in a number of disease conditions [16-20], its importance in the ICU has only been systematically assessed in one study reported from a single medical ICU in a tertiary care university hospital [21].

The importance of excluding patients external to the base population in observational studies in the critically ill has not been well defined. Furthermore, few population-based studies have been conducted among the critically ill and none in the English-language literature have assessed the overall burden and risk factors associated with ICU admission. The objective of this study was to evaluate the impact of inclusion of nonresidents in population-based studies on the occurrence of, on the risk factors for, and on the outcomes of ICU admission.

## Materials and methods

### Study population

The Calgary Health Region (CHR) administers all medical and surgical acute care to the residents of the cities of Calgary and Airdrie, and to approximately 20 nearby small towns, villages, and hamlets (2001 population, 958,610). In April 2003 the CHR was expanded to include the adjacent mountain parks and Wheatland regions [22]. All tertiary care services are provided by the CHR with the only exception being liver, heart, or lung transplantation, where patients are referred to the provincial program in Edmonton. The acute care institutions within the CHR also serve as referral centers for other communities in southern Alberta and the neighboring provinces of British Columbia and Saskatchewan.

All adult ICUs within the CHR are closed units staffed by fully trained intensivists, and they are administered by the Department of Critical Care Medicine, University of Calgary and the CHR. These ICUs currently include a 14-bed cardiovascular surgery intensive care unit (CVICU) and a 22-bed multidisciplinary ICU that serves as the regional trauma and neurosurgical referral center at the Foothills Medical Centre, a 12-bed multidisciplinary ICU at the Peter Lougheed Centre that is also the vascular surgery referral center, and a 10-bed multidisciplinary ICU at the Rockyview General Hospital. All patients 18 years and older admitted to an adult multidisciplinary ICU or the CVICU in the CHR between 1 May 1999 and 30 April 2003 were included. Ethics approval was obtained from the Conjoint Health Research Ethics Board at the University of Calgary and the CHR.

### Protocol

The study utilized a population-based surveillance cohort design with linkage of data collected from regional critical care and administrative databases. Demographic data, clinical data, basic laboratory data, and scoring data were obtained from all patients admitted to ICUs in the CHR in a consistent manner across all sites using the ICU Tracer database, as previously described [23,24]. Patients were classified as CHR residents or nonresidents using data from the CHR Data Warehouse (a regional administrative database), where regional residents are flagged if their home address is within the geographical boundaries of the CHR.

Severity of illness at admission was assessed using the Acute Physiology and Chronic Health Evaluation (APACHE) II score, and the intensity of care was assessed using the Therapeutic Intervention Scoring System score [25,26]. Shock was defined as a mean arterial pressure < 60 mmHg on the first day of admission to the ICU or requirement for a vasopressor infusion. The diagnosis of systemic inflammatory response syndrome (SIRS) was based on a modification of consensus criteria and required at least two of the following; heart rate > 90/min, respiratory rate > 20/min, temperature < 36°C or > 38°C, or white blood cell count < 4 × 10^9^/l or > 12 × 10^9^/l [15]. A surgical patient was any patient recorded as having an operative diagnosis or any patient admitted from the trauma ward or directly from the operating room.

### Statistical analysis

Analysis was performed using Stata version 8.0 (Stata Corp, College Station, TX, USA). With the exception of calculating SIRS criteria, where missing values were treated as normal, missing data were not replaced and a reduced number (*n*) reported where they ocurred. Only first ICU presentations were analyzed from patients with multiple ICU admissions. Normally or near-normally distributed variables were reported as means ± standard deviations and non-normally distributed variables were reported as medians with interquartile ranges (IQRs). Means were compared using the Student *t *test and medians were compared using the Mann–Whitney *U *test. Differences in proportions among categorical data were assessed using Fisher's exact test. Incidence rates were calculated using regional denominator data and compared as previously described [2]. Levels of significance were not *a priori *adjusted for multiple testing, and a two-sided *P *< 0.05 was considered significant for all comparisons.

A multivariable logistic regression model was developed to assess independent risk factors for death. The initial model included clinically suspected variables and those identified as potentially important predictors, including CHR residency, multidisciplinary ICU admission as compared with CVICU admission, the presence of SIRS, shock, hypothermia, age, gender, surgical diagnosis, and APACHE II and Therapeutic Intervention Scoring System scores. Backward stepwise variable elimination was then performed to develop the final model. The final model discrimination was assessed using the area under the receiver operator curve and calibration using the Hosmer–Lemeshow goodness-of-fit test.

## Results

During the 4-year study period 12,193 adult patients had a total of 13,638 admissions to CHR ICUs; 4509 were surgical admissions for less than 48 hours. Overall 7767 (63.7%) patients were classified as CHR residents, for an incidence of ICU admission of 263.7 per 100,000 per year. Both the quarterly and yearly numbers of admissions were stable over the study. More than one-third (4426) of patients were nonresident in the CHR (incidence not able to be calculated) and were primarily (3424 patients) from other health regions in Alberta, 705 patients were from British Columbia, 121 were from Saskatchewan, 135 were from other Canadian provinces and territories, and 41 were international residents. Among the four study ICUs there were 4715 admissions to the CVICU, 3584 to Foothills Medical Centre ICU, 2144 to Peter Lougheed Centre ICU, and 1750 to Rockyview General Hospital ICU, of which 2587 (54.9%), 2264 (63.2%), 1541 (71.9%), and 1375 (78.6%) were CHR residents, respectively. A significant proportional difference in admission rates for CHR and non-CHR residents was observed (*P *< 0.001) between each of the ICUs.

### Demographic features

**Figure 1:**
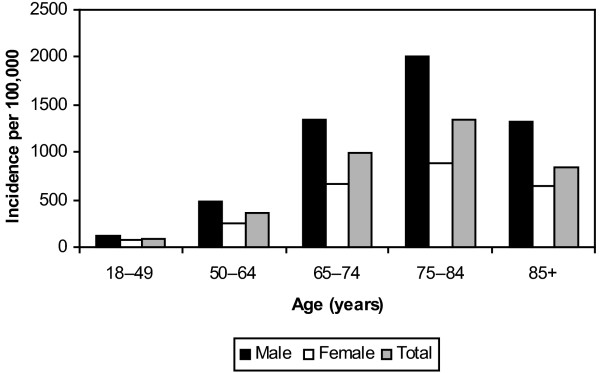
Age-specific and gender-specific population incidence of intensive care unit admission in Calgary Health Region, Alberta, Canada.

The overall median age (IQR) was 64.6 years (50.6–74.0 years) and 7819 patients (64.1%) were male. Although the overall median age of CHR and non-CHR residents was not different, in the subgroup of patients aged 85 years and older patients were nearly twice as likely to be CHR residents (relative risk [RR], 1.80; 95% confidence interval [CI], 1.43–2.27; *P *< 0.0001). There was a gender difference associated with residency status as non-CHR residents were significantly more likely to be male as compared with CHR residents (67.9% versus 62.0%; *P *< 0.0001). Age-specific and gender-specific population incidence rates were established for the population-based cohort as shown in Fig. 1. Males were at significant increased risk for ICU admission as compared with females (330.5 per 100,000 versus 198.2 per 100,000; RR, 1.67; 95% CI, 1.59–1.74; *P *< 0.0001), and this was consistent observed among all age groups (Fig. 1). Increasing risk was associated with incrementally advancing age up to the age of 85 years, where a decrease in incidence was then observed (Fig. 1). As compared with younger individuals, those aged 65 years and older were at substantially increased risk of admission to an ICU (1719.9 per 100,000 versus 238.7 per 100,000; RR, 7.21; 95% CI, 6.95–7.47; *P *< 0.0001).

### Clinical features

Although the magnitudes of differences were small, a number of clinical features were significantly different among CHR and non-CHR residents, as presented in Table 1. In general, non-CHR residents had more markers of increased severity as compared with CHR residents (Table 1). No difference was observed between CHR and non-CHR residents in the occurrence of SIRS, although overall 90% (11,020) of patients fulfilled criteria.

### Outcomes

The overall medians of ICU length of stay and hospital length of stay were 1.9 (IQR, 1–3.9) and 11 (IQR, 6–21), respectively. No significant differences were observed between CHR and non-CHR residents with respect to length of stay. In total, 1443 (11.8%) patients died in the ICU and a further 667 died during that hospitalization, for an overall inhospital case fatality rate of 17.3%. There was a significant effect of CHR residency on case fatality; CHR residents were much more likely to die in the ICU (1016 [13.1%] versus 427 [9.6%]; RR, 1.36; 95% CI, 1.22–1.51; *P *< 0.0001) and in hospital (1547 [20%] versus 563 [12.7%]; RR, 1.57; 95% CI, 1.43–1.71; *P *< 0.0001) as compared with non-CHR resident patients. A multivariable logistic regression model (*n *= 11,569) was developed that had good fit (*P *= 0.4) and discrimination (area under receiver operator curve = 0.83). As presented in Table 2, CHR residency status was independently associated with inhospital death.

## Discussion

This study describes the occurrence of, the demographic risk factors for, and the outcome associated with ICU admission in a large nonselected North American population. Although it is notable that the annual incidence of ICU admission is reported, it is of greater interest that demographic risk groups in the population that were at increased risk for admission to an ICU were defined. Not surprisingly, older age and male gender were associated with an increased need for ICU admission. This may be at least partly due to a higher rate of comorbid conditions, such as smoking or alcohol use, or other high-risk behaviors or activities among males as compared with females [4,5]. The population-based cohort design is an excellent method for defining the actual magnitude of such risks [2]. However, detailed information on each of the patient's comorbidities was not available for all patients in this study, and as a result the risk factor analysis was limited to the evaluation of demographic features alone. The actual burden of disease requiring ICU admission in an entire population was established in a minimally biased fashion in this study. Such accurate information on the degree of human suffering and death related to critical illness is important to potentially support continued or increased funding of clinical ICUs and critical care medical research.

This study demonstrates that inclusion of nonresidents of a base population may have a major impact on biasing the results of studies in the ICU. When nonresidents of the CHR were included in this study, the occurrence of ICU admission in the CHR was overestimated by more than 50%. This observation is consistent with previous studies in the CHR and elsewhere in noncritically ill specific populations [2,16,19,27]. On the other hand, it is highly unlikely that a significant number of CHR residents requiring ICU admission were missed in this study. This is because all multidisciplinary and cardiovascular surgical ICUs in the CHR were included in surveillance and that, with the exception of acute liver, heart, and lung transplantation, patients are rarely referred out of the CHR for provision of healthcare. Furthermore, the CHR is relatively geographically isolated, with the closest tertiary care center to the CHR in Edmonton approximately 300 km away. Therefore, with the exception of the small number of CHR residents who may have required ICU admission while traveling, it seems unlikely that a substantial number of CHR residents requiring ICU admission would have been lost to analysis in this study.

A number of statistically significant differences in the clinical features between CHR and non-CHR residents (Table 1) were observed, and with the exception of fever these would remain significant even if a conservative correction for multiple statistical comparisons such as the Bonferroni method were used. However, although statistically significant, the magnitudes of these differences are small and may not be of practical clinical difference. On the other hand, there was a dramatic effect of residency status on the outcome of patients admitted to ICUs in the CHR. The observation of a lower mortality among non-CHR patients is in contrast to the recent hospital-based study reported by Rosenberg and colleagues, although the definition of 'referral' was different in their study [21]. The reason why non-CHR patients were at lower risk for inhospital mortality is unexplained by the present study data, especially given that they appeared in general to be somewhat sicker on average than CHR residents (Table 1). The possibility exists that non-CHR patients may have died after transfer back to their 'home' health region hospitals and have therefore not been captured in the study inhospital mortality. This would explain the apparent lower inhospital case fatality rate among non-CHR residents but is only speculation. Of note, there were no significant pair-wise interactions between residency status and each of the other variables in the multivariable model. This study demonstrates that if nonresidents of a base population are included in studies of patients admitted to ICUs, gross errors in the determination of occurrence and outcomes may occur.

The results of this study raise concerns regarding the generalization of results obtained from hospital-based reviews or population-based studies where nonresidents are included. However, this may not always be of major practical significance depending on specific study objectives. For example, a hospital-based study defining the outcome of a certain patient population such as transplant patients may be generalizable to other transplant centers because transplant recipients are nearly always managed at academic tertiary care referral institutions [28]. Generalization of results to other populations may therefore not be necessary. Similarly, population-based studies that strictly exclude nonresidents may not always be necessary for providing important information to guide allocation of health resources at regional levels. For example, Manns and colleagues conducted an economic evaluation of activated protein C for severe sepsis using clinical information from such a 'population-based' cohort in the CHR [29]. Although they included non-CHR residents, their results should be widely generalizable to other centers in North America and worldwide because typically patients requiring this therapy are admitted in tertiary care ICUs that are composed of a substantial number of referral patients. It should be recognized, however, that although studies that suffer from such selection bias may provide useful clinical information, results should not be generalized to unlike patient cohorts, and rarely, if ever, to the population as a whole.

There are some limitations to this study that merit discussion. First, the CHR may have a different socioeconomic and demographic profile as compared with other regions, and this may influence the validity of generalizing results to other populations. One advantage, however, is that since this study was population-based, age and gender standardization against a reference population may be performed to facilitate comparison among different regions. This has been demonstrated to be of significant value in other population-based studies conducted in the United States [3]. Second, although the data were collected in a uniform fashion at each of the regional ICUs and much of this was directly linked from bedside monitors, systematic manual auditing of the information was not performed. However, previous work has suggested a high degree of accuracy [24]. Third, the need for admission to an ICU in this study was determined by the attending intensivist and not on some predefined objective criteria. This may be important for generalization to other centers that use different criteria for ICU admission. For example, patients admitted to Canadian ICUs tend to be sicker than those admitted to American ICUs, although adjustment according to APACHE II scores is possible [30]. Fourth, we did not have adequate admission data to further define patients into more refined subgroups for analysis. Finally, it is possible that some case patients were missed by our study surveillance as a result of care external to the CHR. However, given the comprehensiveness of the critical care system in the CHR and its relative geographic isolation, this would be only expected to have a minor effect on the study findings.

## Conclusion

This study demonstrates the adverse effect of inclusion of nonresident patients of the base population on the determination of occurrence and outcome in studies of patients admitted to ICUs. Further well-designed, population-based studies in other regions that exclude nonresidents of the base population are required to better define the distribution and determinants of ICU admission internationally.

## Key messages

• 	This population-based cohort study included all adults admitted to CHR multidisciplinary and cardiovascular surgical ICUs during a 4-year period. The effect of inclusion of non-residents in the study was evaluated.

• 	Failure to exclude non-residents would lead to an overestimation of the incidence of ICU admission by more than 50%. A number of clinical features were significantly different between resident and non-resident patients; most notably, the in-hospital mortality rate was much lower in the non-resident cohort.

• 	This study supports that non-resident patients should be strictly excluded from population-based studies.

## Competing interests

The authors declare that they have no competing intrests.

## Abbreviations

APACHE = Acute Physiology and Chronic Health Evaluation; CHR = Calgary Health Region; CI = confidence interval; CVICU = cardiovascular surgery intensive care unit; ICU = intensive care unit; IQR = interquartile range; RR = relative risk; SIRS = systemic inflammatory response syndrome.

## Figures and Tables

**Table 1:** Statistically significant different clinical features of Calgary Health Region residents and nonresidents admitted to intensive care units, Alberta, Canada

Characteristic	Calgary Health Region resident	Non-Calgary Health Region resident	Total	*P *value
APACHE II score (mean ± standard deviation)	24.90 ± 8.71 (*n *= 7704)	25.46 ± 8.16 (*n *= 4381)	25.10 ± 8.52 (*n *= 12,085)	< 0.001
APACHE II score = 25	3556/7704 (53.8%)	1818/4381 (58.5%)	5374/12,085 (55.5%)	< 0.0001
TISS score (mean ± standard deviation)	43.52 ± 18.78 (*n *= 7411)	48.94 ± 17.99 (*n *= 4260)	45.50 ± 18.67 (*n *= 11,671)	< 0.0001
Surgical patient	4600/7754 (59%)	3254/4420 (74%)	7854/12,174 (65%)	< 0.0001
Fever = 37.8°C	3683/7604 (48.4%)	2200/4343 (50.7%)	5883/11,947 (49.2%)	0.02
Hypothermia < 35°C	1413/7610 (18.6%)	950/4347 (21.9%)	2363/11,957 (19.8%)	< 0.0001
Shock	4551/7767 (58.6%)	2731/4426 (61.7%)	7282/12,193 (59.7%)	< 0.001
Tachycardia = 100/min	5059/7698 (65.7%)	2729/4381 (62.3%)	7788/12,079 (64.5%)	< 0.001
Median (interquartile range) respiratory rate/min	26 (20–33)	24 (18–30)	25 (19–32)	<0.0001

APACHE, Acute Physiology and Chronic Health Evaluation; TISS, Therapeutic Intervention Scoring System.

**Table 2:** Multivariable logistic regression modeling of risk factors for inhospital death among patients admitted to intensive care units in the Calgary Health Region, Alberta, Canada

Variable	Odds ratio	95% Confidence interval^a^
APACHE II score = 25	3.06	2.68–3.50
TISS score = 45	2.02	1.75–2.34
Age = 65 years	1.95	1.73–2.20
Hypothermia < 35°C	1.99	1.71–2.32
Shock	1.66	1.46–1.88
Calgary Health Region resident	1.36	1.20–1.55
Noncardiac surgery^b^	0.57	0.50–0.64
Cardiac surgery^b^	0.02	0.02–0.03

APACHE, Acute Physiology and Chronic Health Evaluation; TISS, Therapeutic Intervention Scoring System.

^a^*P *< 0.0001 for all variables.

^b^As compared with medical diagnosis as the reference group.
